# Determinants of Nucleotide-Binding Selectivity of Malic Enzyme

**DOI:** 10.1371/journal.pone.0025312

**Published:** 2011-09-29

**Authors:** Ju-Yi Hsieh, Meng-Chun Chen, Hui-Chih Hung

**Affiliations:** 1 Department of Life Sciences, National Chung Hsing University, Taichung, Taiwan; 2 Institute of Genomics and Bioinformatics, National Chung Hsing University, Taichung, Taiwan; 3 Agricultural Biotechnology Center, National Chung Hsing University, Taichung, Taiwan; University of South Florida College of Medicine, United States of America

## Abstract

Malic enzymes have high cofactor selectivity. An isoform-specific distribution of residues 314, 346, 347 and 362 implies that they may play key roles in determining the cofactor specificity. Currently, Glu314, Ser346, Lys347 and Lys362 in human c-NADP-ME were changed to the corresponding residues of human m-NAD(P)-ME (Glu, Lys, Tyr and Gln, respectively) or *Ascaris suum* m-NAD-ME (Ala, Ile, Asp and His, respectively). Kinetic data demonstrated that the S346K/K347Y/K362Q c-NADP-ME was transformed into a debilitated NAD^+^-utilizing enzyme, as shown by a severe decrease in catalytic efficiency using NADP^+^ as the cofactor without a significant increase in catalysis using NAD^+^ as the cofactor. However, the S346K/K347Y/K362H enzyme displayed an enhanced value for *k*_cat,NAD_, suggesting that His at residue 362 may be more beneficial than Gln for NAD^+^ binding. Furthermore, the S346I/K347D/K362H mutant had a very large *K*_m,NADP_ value compared to other mutants, suggesting that this mutant exclusively utilizes NAD^+^ as its cofactor. Since the S346K/K347Y/K362Q, S346K/K347Y/K362H and S346I/K347D/K362H c-NADP-ME mutants did not show significant reductions in their *K*_m,NAD_ values, the E314A mutation was then introduced into these triple mutants. Comparison of the kinetic parameters of each triple-quadruple mutant pair (for example, S346K/K347Y/K362Q versus E314A/S346K/K347Y/K362Q) revealed that all of the *K*_m_ values for NAD^+^ and NADP^+^ of the quadruple mutants were significantly decreased, while either *k*_cat,NAD_ or *k*_cat,NADP_ was substantially increased. By adding the E314A mutation to these triple mutant enzymes, the E314A/S346K/K347Y/K362Q, E314A/S346K/K347Y/K362H and E314A/S346I/K347D/K362H c-NADP-ME variants are no longer debilitated but become mainly NAD^+^-utilizing enzymes by a considerable increase in catalysis using NAD^+^ as the cofactor. These results suggest that abolishing the repulsive effect of Glu314 in these quadruple mutants increases the binding affinity of NAD^+^. Here, we demonstrate that a series of E314A-containing c-NADP-ME quadruple mutants have been changed to NAD^+^-utilizing enzymes by abrogating NADP^+^ binding and increasing NAD^+^ binding.

## Introduction

Malic enzyme catalyzes a reversible oxidative decarboxylation that converts L-malate into CO_2_ and pyruvate with the simultaneous reduction of NAD(P)^+^ to NAD(P)H [1]–[3] and requires a divalent metal ion (Mn^2+^ or Mg^2+^) for catalysis. The enzyme is widely distributed in nature, with conserved sequences and similar tertiary structural topologies among different species [4]–[7]. Mammalian malic enzymes are classified into three isoforms according to their cofactor specificities and subcellular localizations: cytosolic NADP^+^-dependent (c-NADP-ME) [8], [9], mitochondrial NADP^+^-dependent (m-NADP-ME) [10], and mitochondrial NAD(P)^+^-dependent malic enzymes (m-NAD(P)-ME) [11]–[13]. The m-NAD(P)-ME isoform can use either NAD^+^ or NADP^+^ as a cofactor, but the enzyme favors NAD^+^ as the physiological cofactor [11], [14]. Furthermore, this enzyme isoform is an allosteric enzyme [15], [16] that can be inhibited by ATP [17], [18]. Malic enzyme has a specific role in cells. Both c-NADP-ME and m-NADP-ME act as NADPH suppliers during the biosynthesis of long-chain fatty acids and steroids, so they are defined as lipogenic enzymes [1], [19], [20]. Human m-NAD(P)-ME is believed to play an important role in the metabolism of glutamine in fast-growing tissues and tumors [21]–[23]. Therefore, m-NAD(P)-ME is regarded as a potential target for cancer therapy.

**Figure 1:**
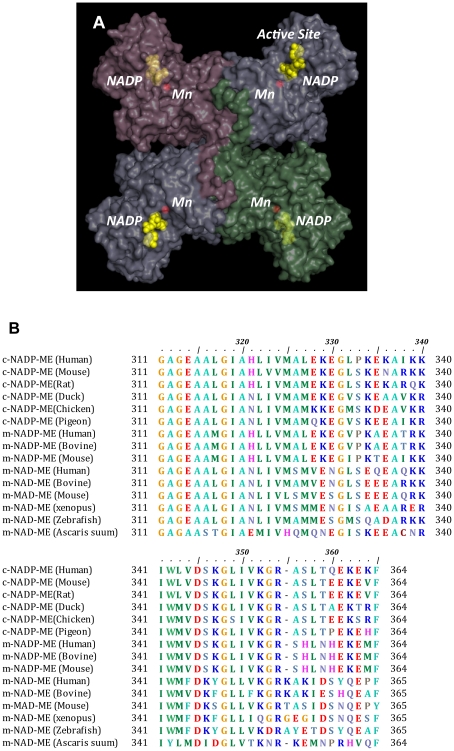
Nucleotide-binding site of c-NADP-ME. (**A**) Tetramer of pigeon c-NADP-ME (PDB code 1GQ2). The active site with Mn^2+^ and NADP^+^ in each subunit is indicated. NADP^+^ in the active site is colored yellow, and the Mn^2+^ ion is red; they are displayed in the sphere model. (**B**) Multiple sequence alignments of three clusters of malic enzyme isoforms around the nucleotide-binding region of the active site. Amino acid sequences of malic enzymes were searched using BLAST [34], and the alignments were generated by Clustal W [35]. This figure was generated using the BioEdit sequence alignment editor program [36]. (**C**) The binding mode of NADP^+^ in the active site of c-NADP-ME (PDB code 1GQ2). Ser346, Lys347 and Lys362 are colored green, purple and pink, respectively, and they are shown in a ball-and-stick model. The yellow dashed lines represent the polar contacts between amino acid residues and NADP^+^. These figures were generated using PyMOL (DeLano Scientific LLC, San Carlos, CA).

The crystal structures of malic enzyme in complex with its substrate, cofactor, inhibitor and regulator have been solved [5], [6], [24]–[28]. Structural data reveal malic enzyme to be a homotetramer with a dimer-of-dimers quaternary structure (Figure 1A); each monomer contains an active site. The dimer interface formed by subunits A and B (or by C and D) displays more intimate contact than the tetramer interface formed by subunits A and D (or by B and C). Structural data regarding the binary complexes (NAD^+^) [7], ternary complexes (NAD^+^ and Lu^3+^) [26], quaternary complexes (NAD^+^, substrate analog inhibitors and divalent cation) [24] and pentary complexes (NAD^+^/NADH, substrate malate/pyruvate, divalent cation, and allosteric activator fumarate) [27], [28] of human m-NAD(P)-ME reveal that the enzyme may exist either in open forms or closed forms and that it may undergo an open-closed transition during catalysis [4], [24]. The structure of pigeon c-NADP-ME in quaternary complexes with NADP^+^, oxalate, and a divalent ion is in a closed form [6]. However, the structure of the open form for the cytosolic isoform has not yet been determined. Furthermore, c-NADP-ME is not a cooperative or allosteric enzyme, and it is less inhibited by ATP [29]. Thus, it appears to lack the allosteric and exo sites that are present in m-NAD(P)-ME.

The nucleotide-binding regions of malic enzyme isoforms are different. Crystal structures of the quaternary complexes of pigeon c-NADP-ME reveal that the 2′-phosphate group of NADP^+^ interacts with Ser346 and the ammonium group of Lys362 [6]. Previous studies on pigeon c-NADP-ME suggest that NADP^+^ specificity is determined by an electrostatic interaction between the ε-amino group of Lys362 and the 2′-phosphate of NADP^+^ [30]. Moreover, kinetic studies on human m-NAD(P)-ME demonstrate that the K346S/Y347K/Q362K substitutions in human m-NAD(P)-ME cause it to be a NADP^+^-dependent enzyme [14], [31]. Lys346 in human m-NAD(P)-ME has minor effects on cofactor preference but has a significant impact on isoform-specific ATP inhibition [29]. Tyr347 is also a determinant of the dual-cofactor specificity of human m-NAD(P)-ME [31]. Multiple sequence alignments reveal an isoform-specific distribution of residues 346, 347 and 362, which are Ser, Lys and Lys, respectively, in NADP^+^-dependent malic enzyme and Ile, Asp and His, respectively, in NAD^+^-dependent malic enzyme (Figure 1B), implying that they may play key roles in determining the cofactor specificity of malic enzyme. In addition, our previous studies showed that Glu314 may be involved in the binding affinities of NAD^+^ and ATP [18]. In the present study, Glu314, Ser346, Lys347 and Lys362 in human c-NADP-ME were changed to the corresponding residues of human m-NAD(P)-ME and *Ascaris suum* m-NAD-ME. Here, we provide full kinetic evidence to reveal the determinants that govern the nucleotide-binding selectivity of malic enzyme.

## Results

### Kinetic properties of human recombinant c-NADP-ME

Kinetic parameters of c-NADP-ME determined using NADP^+^ or NAD^+^ as the cofactor (*K*_m,NADP_ and *K*_m,NAD_) are shown in Table 1. For the wild-type (WT) enzyme, the *K*_m,NADP_ and *K*_m,NAD_ values of human c-NADP-ME were 0.0035 mM and 18.57 mM, respectively, revealing that the enzyme had a much higher apparent affinity for NADP^+^ than NAD^+^. Furthermore, the *k*_cat,NADP_ value (126 s^−1^) of the human c-NADP-ME was higher than the *k*_cat,NAD_ value (51 s^−1^). The *k*_cat,NADP_/*K*_m,NADP_ and *k*_cat,NAD_/*K*_m,NAD_ values of the enzyme were 3.6×10^7^ and 2.8×10^3^ M^−1^s^−1^, respectively, a 10^4^-fold difference.

**Table 1:** Kinetic parameters for human wild-type and nucleotide-binding mutant variants of c-NADP-ME.

c-NADP-ME	*K*_m,NADP_ (mM)	Fold increase *K*_m(NADP)_	*K*_m,mal(NADP)_ (mM)	Fold increase *K*_m,mal(NADP)_	*k*_cat,NADP_ (s^−1^)	*k*_cat,NADP_ ratio relative to WT	*K*_m,NAD_ (mM)	Fold decrease *K*_m(NAD)_	*K*_m,mal(NAD)_ (mM)	Fold decrease *K*_m,mal(NAD)_	*k*_cat,NAD_ (s^−1^)	*k*_cat,NAD_ ratio relative to WT
**WT**	0.005±0.001	1	0.9±0.01	1	146±4	1	19±4	1	5±0.5	1	51±5	1
**E314A**	0.002±0.0004	0.4	0.4±0.1	0.4	110±2	0.75	1.6±0.3	12	4±0.8	1	113±5	2.2
**S346K**	0.013±0.001	2.6	0.8±0.1	0.9	129±3	0.88	17±2	1.1	4±0.2	1.1	22±1	0.4
**E314A/S346K**	0.003±0.0003	0.58	1±0.1	1.2	137±2	0.94	5±0.7	3.6	4±0.3	1.1	145±7	2.8
**K347Y**	0.03±0.001	5.4	2±0.1	1.8	121±2	0.83	11±3	1.7	5±0.3	0.9	40±4	0.8
**K362Q**	0.7±0.1	144	2±0.1	1.8	132±3	0.90	13±2	1.5	5±0.3	1.0	60±4	1.2
**K362H**	0.3±0.02	58.	4±0.3	4.9	149±2	1.02	14±2	1.4	8±0.3	0.6	108±7	2.1
**S346K/K347Y**	0.14±0.02	28	1±0.1	1.0	102±2	0.70	18±1	1.1	5±0.3	0.9	65±2	1.3
**S346K/K362Q**	6±0.5	1194	3±0.3	3.7	112±3	0.77	14±2	1.4	4±0.3	1.3	110±5	2.2
**K347Y/K362Q**	12±0.5	2400	5±0.1	5.5	78±2	0.53	20±2	1	8±0.4	1	53±2	1.0
**S346K/K347Y/K362Q**	17±2	3400	9±1	10	15±1	0.10	10±1	1.9	4±0.1	1.4	124±5	2.4
**E314A/S346K/K347Y/K362Q**	5±1.5	900	5±0.7	5.6	51±8	0.35	1.5±0.2	13	2±0.3	3	208±6	4.1
**S346K/K347Y/K362H**	29±7	5700	10±3	11	8±1.2	0.05	7.1±0.7	2.7	6±0.8	0.9	219±9	4.3
**E314A/S346K/K347Y/K362H**	3±0.5	620	7±0.3	7.3	26±1	0.18	0.9±0.2	21	1±0.2	4	258±2	5.1
**S346I/K347D/K362H**	116±102	23260	32±5	36	1.3±0.1	0.01	6.5±0.8	2.9	7±0.8	0.8	131±8	2.6
**E314A/S346I/K347D/K362H**	29±10	5806	21±3	24	15±3	0.10	0.9±0.1	21	3±0.5	2	166±5	3.3

Most of the human mutant c-NADP-ME variants listed in Table 1 showed notably increased *K*_m,NADP_ values but exhibited relatively minor changes in *K*_m,NAD_ values. The K362Q enzyme displayed a significant (over 140-fold) elevation in *K*_m,NADP_ value compared with that of WT c-NADP-ME but no significant changes in the *k*_cat,NADP_ value, again showing that Lys362 is the key residue contributing to binding the 2′-phosphate of NADP^+^. In *Ascaris suum* m-NAD-ME, residue 362 is His (Figure 1B). The K362H enzyme also displayed a considerable elevation in *K*_m,NADP_, but the *k*_cat,NAD_ value was elevated compared to the WT and K362Q c-NADP-ME, indicating that His362 may be advantageous for NAD^+^-specific catalysis in NAD-ME. The S346K and K347Y enzyme variants showed a 3- to 5-fold increase in *K*_m,NADP_ compared to the WT enzyme, and the *K*_m,NADP_ value of the double mutant S346K/K347Y further increased 30-fold. Furthermore, the *K*_m,NADP_ values of the double mutants S346K/K362Q and K347Y/K362Q were much higher than that of the single mutant K362Q and more than several thousand times higher than that of WT c-NADP-ME. These results suggest that both Ser346 and Lys347 represent additional factors that determine the NADP^+^ specificity of human c-NADP-ME. Indeed, both double-mutant enzymes, S346K/K362Q and K347Y/K362Q, displayed dual-cofactor specificity, as both of them exhibited similar values for *K*_m,NADP_ and *K*_m,NAD_ and for *k*_cat,NADP_ and *k*_cat,NAD_. For S346K/K347Y/K362Q, the *K*_m,NADP_ value was elevated to 16.9 mM, which was approximately 3,400 times greater than that of the WT c-NADP-ME. At the same time, the *k*_cat,NADP_ of this triple mutant was reduced to only 10% of WT, and the *K*_m,NAD_ value decreased about twofold, with a twofold increase in *k*_cat,NAD_, indicating that this mutant was transformed into an NAD^+^-favoring enzyme. The *k*_cat,NADP_/*K*_m,NADP_ and *k*_cat,NAD_/*K*_m,NAD_ values of this triple-mutant enzyme were 8.8×10^2^ and 1.2×10^4^ M^−1^s^−1^, respectively.

Comparing the kinetic parameters of S346K/K362Q and S346K/K347Y/K362Q, the *K*_m,NADP_ value of the triple mutant was slightly elevated with a significant decrease in *k*_cat,NADP_. However, the *K*_m,NAD_ and *k*_cat,NAD_ values were not significantly changed by adding the K347Y mutation to the S346K/K362Q enzyme. In contrast, the major differences in kinetic parameters between K347Y/K362Q and S346K/K347Y/K362Q were that the *k*_cat,NADP_ value was further reduced and that the *k*_cat,NAD_ value was increased significantly by adding the S346K mutation to the K347Y/K362Q enzyme. These results indicate that the K347Y mutation contributed considerably to the decrease in *k*_cat,NADP_, whereas the S346K mutation had a significant effect on the increase in *k*_cat,NAD_ with a concurrent decrease in *k*_cat,NADP_.

The S346K/K347Y/K362Q c-NADP-ME displayed an elevated *K*_m,NADP_ value, but with no concurrent reduction in its *K*_m,NAD_ value, revealing that the mutation of Ser346 to Lys, Lys347 to Tyr and Lys362 to Gln significantly reduced the NADP^+^ specificity without increasing its apparent affinity for NAD^+^. However, mutation of Lys362 to His instead of Gln caused the enzyme to have a greater *k*_cat,NAD_ value than the enzyme replaced by Gln at residue 362 [K362H (108 s^−1^) vs. K362Q (60 s^−1^) and S346K/K347Y/K362H (219 s^−1^) vs. S346K/K347Y/K362Q (124 s^−1^)]. These results suggested that residue 362 also determines the specificity of the cofactor used by malic enzyme. In c-NADP-ME, which is NADP^+^-specific, this residue is a positively charged Lys, while this residue in m-NAD-ME is His, which is beneficial for NAD^+^ utilization; in the m-NAD(P)-ME, which has dual-cofactor specificity, this residue is a neutrally charged Gln. Furthermore, mutation of Ser346 to Ile, Lys347 to Asp and Lys362 to His (S346I/K347D/K362H), the respective amino acid residues on *Ascaris suum* m-NAD-ME, causes the enzyme to have a very large *K*_m,NADP_ value. The exclusive use of NAD^+^ by the S346I/K347D/K362H mutant is due to a loss of affinity for NADP^+^ as the cofactor rather than greater affinity for NAD^+^. The *k*_cat,NADP_/*K*_m,NADP_ and *k*_cat,NAD_/*K*_m,NAD_ values of this triple-mutant enzyme were 11.2 and 2.9×10^4^ M^−1^s^−1^, respectively.

Because the S346K/K347Y/K362Q, S346K/K347Y/K362H and S346I/K347D/K362H c-NADP-ME mutants did not show reductions in their *K*_m,NAD_ values, additional factors may be involved in nucleotide-binding affinity. Our previous studies demonstrated that Glu314 may have repulsive effects on NAD^+^ and ATP in human m-NAD(P)-ME [18], as the E314A mutant displayed lower *K*_m,NAD_ and *K*_i,ATP_ values than WT. Glu314 is conserved in most MEs; however, this residue is Ala in *Ascaris suum* m-NAD-ME (Figure 1B). Structural studies of the *Ascaris* and human ME⋅NAD binary complexes revealed that residue 314 may interact with the bisphosphate of the NAD moiety [5]. To further investigate whether Glu314 is an influential factor in nucleotide binding, the quadruple mutants E314A/S346K/K347Y/K362Q, E314A/S346K/K347Y/K362H and E314A/S346I/K347D/K362H of c-NADP-ME were created. Comparison of the kinetic parameters of each triple-quadruple mutant pair (for example, S346K/K347Y/K362Q versus E314A/S346K/K347Y/K362Q) revealed that all of the *K*_m_ values for NAD^+^ and NADP^+^ of the quadruple mutants were significantly decreased, while either *k*_cat,NAD_ or *k*_cat,NADP_ was substantially increased (Table 1). We believed that the significant change in these kinetic parameters, especially for the binding of NAD^+^, was caused by the mutation of Glu314 to Ala. The *K*_m,NAD_ was drastically decreased and the *k*_cat,NAD_ value was increased for the E314A single mutant (Table 1). By adding the E314A mutation to these triple mutant enzymes, their *K*_m,NAD_ values were reduced nearly 10-fold (Table 1).

Human m-NAD(P)-ME is a non-cooperative enzyme for substrate L-malate binding. Table 1 also lists the *K*_m_ values of L-malate using NADP^+^ or NAD^+^ as the cofactor (*K*_m,mal(NADP)_ and *K*_m,mal(NAD)_). The *K*_m,mal(NADP)_ and *K*_m,mal(NAD)_ values of c-NADP-ME were 0.9 mM and 5.0 mM, respectively. There were no significant differences in *K*_m,mal(NADP)_ for WT, E314A, S346K, E314A/S346K, K347Y, K362Q and S346K/K347Y c-NADP-ME and the value of *K*_m,mal(NADP)_ for K362H, S346K/K362Q, K347Y/K362Q, S346K/K347Y/K362Q, E314A/S346K/K347Y/K362Q, S346K/K347Y/K362H and E314A/S346K/K347Y/K362H were 3- to 10-fold larger than that of WT. However, the *K*_m,mal(NADP)_ value of S346I/K347D/K362H and E314A/S346I/K347D/K362H were 32 mM and 21 mM, respectively, over 20-fold larger than that of WT. In addition, the values of *K*_m,mal(NAD)_ for WT and mutant c-NADP-ME did not display significant differences. Considering these kinetic data together, we can conclude that multiple mutations of E314A, S346K, K347Y and K362Q have profound effects on the enzyme by increasing *K*_m,NADP_ and *K*_m,mal(NADP)_, significantly decreasing *k*_cat,NADP_ and increasing *K*_m,NAD_ and *k*_cat,NAD_; however, these mutations had only minor influences on *K*_m,mal(NAD)_.

### Inhibitory effect of ATP on c-NADP-ME

The enzyme c-NADP-ME is much less sensitive to ATP inhibition than m-NAD(P)-ME [31]. Unlike m-NAD(P)-ME, which is inhibited by ATP with a inhibition constant (*K*_i_) of 0.7–1.2 mM, c-NADP-ME showed less inhibition by ATP with a *K*_i_ value of 5–17 mM [18], [29], [31]. We have reported that Glu314 and Lys346 are influential factors for the ATP sensitivity of human m-NAD(P)-ME [18], [29] and have opposite effects. The E314A human m-NAD(P)-ME is more sensitive to ATP inhibition with a smaller *K*_i,ATP_ value of 0.5 mM [18]. In contrast, human m-NAD(P)-ME enzymes containing the K346S mutation are much less sensitive to ATP inhibition with larger *K*_i,ATP_ values [31].

**Figure 2:**
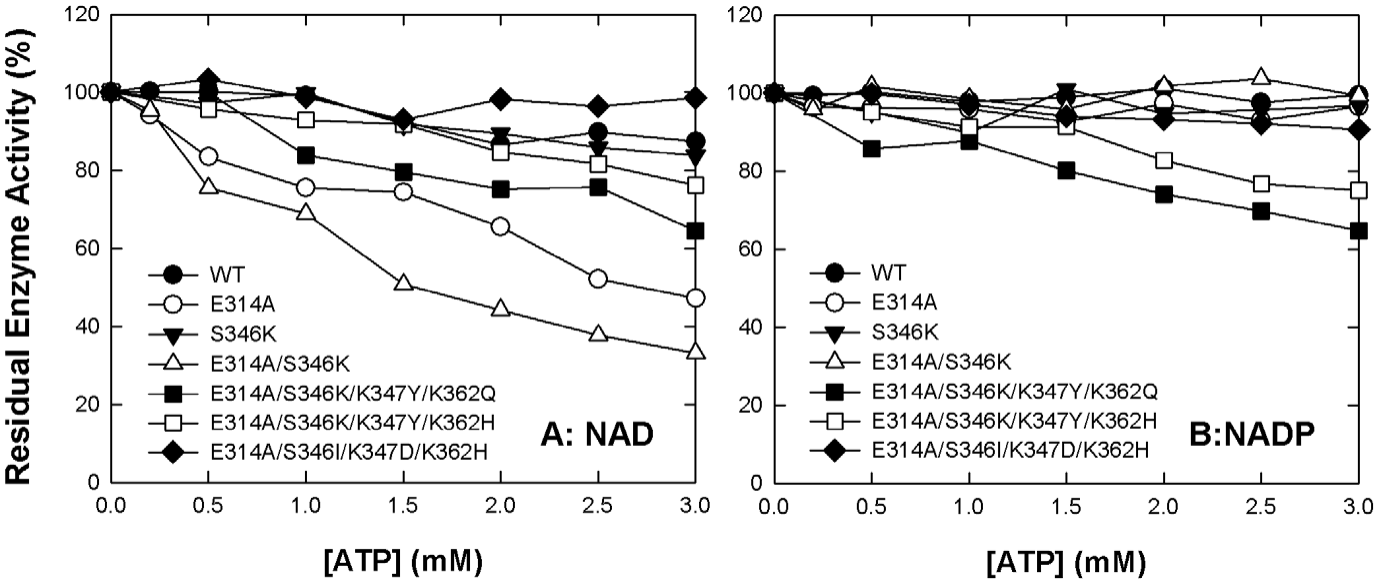
Inhibitory effect of ATP on human WT and mutant c-NADP-ME. The inhibited enzyme activities were assayed with NAD^+^ (**A**) or NADP^+^ (**B**) as the cofactor. The assay mixture contains 40 mM malate, 10 mM MgCl_2_, and 1 mM NAD^+^ or NADP^+^. The ATP concentration ranged from 0 to 3 mM.

Here, we examined the inhibitory effect of ATP on WT and mutant c-NADP-ME and determined the inhibition constants of these enzymes either with NAD^+^ or NADP^+^ as the cofactor, *K*_i,ATP(NAD)_ and *K*_i,ATP(NADP)_. WT c-NADP-ME, with NAD^+^ as the cofactor, showed slight (approximately 10%) inhibition of activity by ATP (Figure 2A, closed circles). The E314A and E314A/S346K enzymes displayed approximately 50% and 70%, respectively, inhibition by ATP (Figure 2A, open circles and triangles, respectively). The quadruple mutants containing both E314A and S346K (E314A/S346K/K347Y/K362Q and E314A/S346K/K347Y/K362H) demonstrated mild ATP inhibition (Figure 2A, open and closed squares, respectively). In contrast, the quadruple mutant E314A/S346I/K347D/K362H was not inhibited by ATP (Figure 2A, closed diamonds) despite containing the E314A substitution. The inhibitory effect of ATP with NADP^+^ as the cofactor was less obvious than when NAD^+^ was the cofactor for these enzymes (Figure 2B).

All of these enzymes presented competitive inhibition patterns either with NAD^+^ or NADP^+^ as the cofactor (data not shown). The ATP inhibition demonstrated a competitive inhibition pattern with a inhibition constant of ATP with respect to NAD^+^ (*K*_i,ATP(NAD)_) for WT c-NADP-ME of 23.2±1.4 mM (Table 2). The E314A and E314A/S346K enzymes demonstrated a competitive inhibition pattern with a much smaller *K*_i,ATP (NAD)_ value of 0.73±0.14 mM and 0.73.±0.11 mM, respectively (Table 2). The *K*_i,ATP(NAD)_ value of the quadruple mutant, E314A/S346K/K347Y/K362Q, was 3.3±0.3 mM (Table 2). The inhibition constant of ATP with respect to NADP^+^ for WT was 20.6±3.0 mM, comparable to that with respect to NAD^+^ (Table 2). Conversely, the *K*_i,ATP(NADP)_ values of E314A and E314A/S346K were 18.1±2.6 mM and 17.4±2.6 mM, respectively, similar to that of WT but larger than that with respect to NAD^+^ (Table 2). The *K*_i,ATP(NADP)_ value of E314A/S346K/K347Y/K362Q was 3.9±0.5 mM, smaller than that of WT and comparable to that with respect to NAD^+^ (Table 2). These inhibition data demonstrate that although the quadruple mutants have significantly reduced their NADP^+^ preference and shifted their cofactor preference toward NAD^+^, their sensitivity to ATP inhibition was not significantly elevated, suggesting that cofactor preference is not associated with inhibition by ATP.

**Table 2:** Inhibition constants of ATP for human c-NADP-ME.

c-NADP-ME	*K*_i,ATP(NAD)_ (mM)	*K*_i,ATP(NADP)_ (mM)
**WT**	23.2±1.4	20.6±3.0
**E314A**	0.73±0.14	18.1±2.6
**E314A/S346K**	0.73±0.11	17.4±2.6
**E314A/S346K/K347Y/K362Q**	3.3±0.3	3.9±0.5

## Discussion

Our previous studies on human m-NAD(P)-ME have suggested that Lys346, Tyr347 and Gln362 together govern the dual-cofactor specificity of the enzyme. Mutation of these residues to the respective amino acid residues in c-NADP-ME (Ser346, Lys347 and Lys362) causes the enzyme to shift its cofactor preference from NAD^+^ to NADP^+^ [31]. In addition, Glu314 in human m-NAD(P)-ME seems to have a repulsive effect on NAD^+^ and ATP [18]. Here, we demonstrate that a series of E314A-containing quadruple-mutant c-NADP-ME variants are changed to NAD^+^-utilizing enzymes by abrogating NADP^+^ binding and increasing the binding of NAD^+^.

Kinetic data demonstrated that the S346K/K347Y/K362Q c-NADP-ME was transformed into a debilitated NAD^+^-utilizing malic enzyme by a severe decrease in catalytic efficiency using NADP^+^ as the cofactor without a significant increase in catalysis using NAD^+^ as the cofactor. However, the S346K/K347Y/K362H enzyme showed enhanced turnover using NAD^+^ as the cofactor (*k*_cat,NAD_, Table 1), suggesting that His at residue 362 may be more beneficial than Gln in NAD^+^ binding. The E314A mutation was then introduced into these triple mutants. For the E314A/S346K/K347Y/K362Q c-NADP-ME, its *k*_cat_ */K*_m,NAD(P)_*K_m,mal_ is 2.3 s^−1^mM^−2^ using NADP^+^ as the cofactor, and 73 s^−1^mM^−2^ using NAD^+^ as the cofactor (Table 3). The fold decrease in *k*_cat,NADP_ */K*_m,NADP_*K_m,mal_ relative to WT is 1.5×10^4^; whereas the fold increase in *k*_cat,NAD_ */K*_m,NAD_*K_m,mal_ is 1.4×10^2^ (Table 3), indicating that the presence of E314A in the E314A/S346K/K347Y/K362Q c-NADP-ME transforms the debilitated triple mutant enzyme into a preferentially NAD^+^-utilizing enzyme; the quadruple mutant displays a considerable increase in catalysis using NAD^+^ as the cofactor. For the E314A/S346K/K347Y/K362H c-NADP-ME, its *k*_cat_ */K*_m,NAD(P)_*K_m,mal_ is 1.3 s^−1^mM^−2^ when using NADP^+^ as the cofactor, and 239 s^−1^mM^−2^ when using NAD^+^ as the cofactor (Table 3). The fold decrease in *k*_cat,NADP_ */K*_m,NADP_*K_m,mal_ relative to WT is 2.5×10^4^; whereas the fold increase in *k*_cat,NAD_ */K*_m,NAD_*K_m,mal_ is 4.5×10^2^ (Table 3). Indeed, this quadruple mutant is the best predominantly NAD^+^-using enzyme in this report, suggesting that elimination of the repulsive effect of Glu314 in these quadruple mutants increases the binding affinity of NAD^+^ (Table 1).

**Table 3:** Specificity constants for human wild-type and nucleotide-binding mutant c-NADP-ME variants.

c-NADP-ME	*k*_cat,NADP_ */K*_m,NADP_*K_m,mal(NADP)_ (s^−1^ mM^−2^)			Fold decrease in *k*_cat,NADP_ */K*_m,NADP_*K_m,mal(NADP)_	*k*_cat,NAD_ */K*_m,NAD_*K_m,mal(NAD)_ (s^−1^ mM^−2^)			Fold increase in *k*_cat,NAD_ */K*_m,NAD_*K_m,mal(NAD)_
**WT**	32809	±	6633	1	0.5	±	0.1	1
**E314A**	137625	±	39042	0.2	19	±	5.3	35
**S346K**	12404	±	1843	2.6	0.3	±	0.04	0.5
**E314A/S346K**	42947	±	5948	0.8	6.2	±	1.0	12
**K347Y**	2733	±	173	12	0.7	±	0.2	1.3
**K362Q**	115	±	9.1	2.9×10^2^	0.9	±	0.2	1.7
**K362H**	117	±	11.4	2.8×10^2^	1	±	0.2	1.8
**S346K/K347Y**	810	±	147	41	0.7	±	0.1	1.3
**S346K/K362Q**	6	±	0.71	5.8×10^3^	2.1	±	0.4	3.9
**K347Y/K362Q**	1.3	±	0.070	2.5×10^4^	0.3	±	0.04	0.6
**S346K/K347Y/K362Q**	0.1	±	0.017	3.4×10^5^	3.4	±	0.4	6.4
**E314A/S346K/K347Y/K362Q**	2.3	±	0.9	1.5×10^4^	73	±	15	1.4×10^2^
**S346K/K347Y/K362H**	0.03	±	0.011	1.2×10^6^	5.3	±	0.9	10
**E314A/S346K/K347Y/K362H**	1.3	±	0.22	2.5×10^4^	239	±	66	4.5×10^2^
**S346I/K347D/K362H**	3.5×10^−4^	±	3.1×10^−4^	9.4×10^7^	3.7	±	0.5	6.8
**E314A/S346I/K347D/K362H**	0.02	±	0.010	1.1×10^6^	59	±	11.8	1.1×10^2^

The S346I/K347D/K362H enzyme displayed very large *K*_m,NADP_ and *K*_m,mal(NADP)_ values with an increased *k*_cat,NAD_ value (Table 1). The *k*_cat_ */K*_m,NAD(P)_*K_m,mal_ of S346I/K347D/K362H c-NADP-ME is only 3.5×10^−4^ s^−1^mM^−2^ when using NADP^+^ as the cofactor; the fold decrease in *k*_cat,NADP_ */K*_m,NADP_*K_m,mal_ relative to WT is 9.4×10^7^ (Table 3), indicating a strong rejection of NADP^+^ by this triple mutant. However, the E314A/S346I/K347D/K362H enzyme displayed less of a bias against NADP^+^ (*K*_m,NADP_). For this quadruple mutant, the *k*_cat_ */K*_m,NAD(P)_*K_m,mal_ is 0.02 s^−1^mM^−2^ when using NADP^+^ as the cofactor, and 59 s^−1^mM^−2^ when using NAD^+^ as the cofactor (Table 3). The fold decrease in *k*_cat,NADP_ */K*_m,NADP_*K_m,mal_ relative to WT is 1.1×10^6^; whereas the fold increase in *k*_cat,NAD_ */K*_m,NAD_*K_m,mal_ is 1.1×10^2^ (Table 3).

### Factors involved in ATP inhibition of c-NADP-ME

The E314A and E314A/S346K c-NADP-ME variants are sensitive to ATP inhibition when using NAD^+^ as the cofactor, but not when using NADP^+^ (Figure 2). These two mutant enzymes, which mainly retain their NADP^+^ selectivity, may be protected by NADP^+^. Thus, ATP is a poor competitive inhibitor with respect to NADP^+^ for the NADP^+^-specific malic enzyme.

The E314A/S346K/K347Y/K362Q, E314A/S346K/K347Y/K362H and E314A/S346K/K347Y/K362H enzymes display altered cofactor specificity from NADP^+^ to NAD^+^. These quadruple mutants with E314A or E314A/S346K mutations, however, are less sensitive to ATP inhibition than the E314A and E314A/S346K c-NADP-ME regardless of whether NADP^+^ or NAD^+^ is used as the cofactor (Figure 2, Table 2). Table 4 summarizes the amino acid identities at residues 314, 346, 347 and 362 for these c-NADP-MEs. The E314A and E314A/S346K c-NADP-ME variants may have a more positively charged nucleotide-binding site with greater affinity for ATP and thus greater sensitivity to ATP inhibition. The quadruple mutants, however, with a less positively charged nucleotide-binding site, may not possess a great enough affinity for ATP.

**Table 4:** Residues involved in ATP inhibition for human wild-type and nucleotide-binding mutant c-NADP-ME variants.

c-NADP-ME	Residue				aATP inhibition (%)	
	314	346	347	362	bNAD^+^	bNADP^+^
**WT**	E	S	K	K	87.3	99.4
**E314A**	A	S	K	K	47.3	96.6
**S346K**	E	K	K	K	83.8	96.8
**E314A/S346K**	A	K	K	K	33.2	99.3
**K347Y**	E	S	Y	K	94.2	96.6
**K362Q**	E	S	K	Q	95.4	99.8
**K362H**	E	S	K	H	88.0	89.3
**S346K/K347Y**	E	K	Y	K	75.2	86.7
**S346K/K362Q**	E	K	K	Q	86.5	91.8
**K347Y/K362Q**	E	S	Y	Q	99.2	91.6
**S346K/K347Y/K362Q**	E	K	Y	Q	71.4	81.3
**E314A/S346K/K347Y/K362Q**	A	K	Y	Q	64.5	64.7
**S346K/K347Y/K362H**	E	K	Y	H	82.9	78.8
**E314A/S346K/K347Y/K362H**	A	K	Y	H	76.2	75.0
**S346I/K347D/K362H**	E	I	D	H	88.5	83.1
**E314A/S346I/K347D/K362H**	A	I	D	H	98.6	90.6

a Residual enzyme activity by inhibition at 3 mM ATP.

b using NAD^+^ or NADP^+^ as the cofactor.

The S346K and E314A c-NADP-ME may have similar positively charged nucleotide-binding sites because the net charges of the sites are apparently equivalent. However, the S346K c-NADP-ME is less sensitive to ATP inhibition than E314A c-NADP-ME. Furthermore, the E314A m-NAD(P)-ME is more sensitive to ATP inhibition than WT m-NAD(P)-ME [18]. These data suggest that Glu314 has opposite effects on ATP binding in m-NAD(P)-ME and c-NADP-ME.

### Nucleotide-binding site of malic enzyme

**Figure 3:**
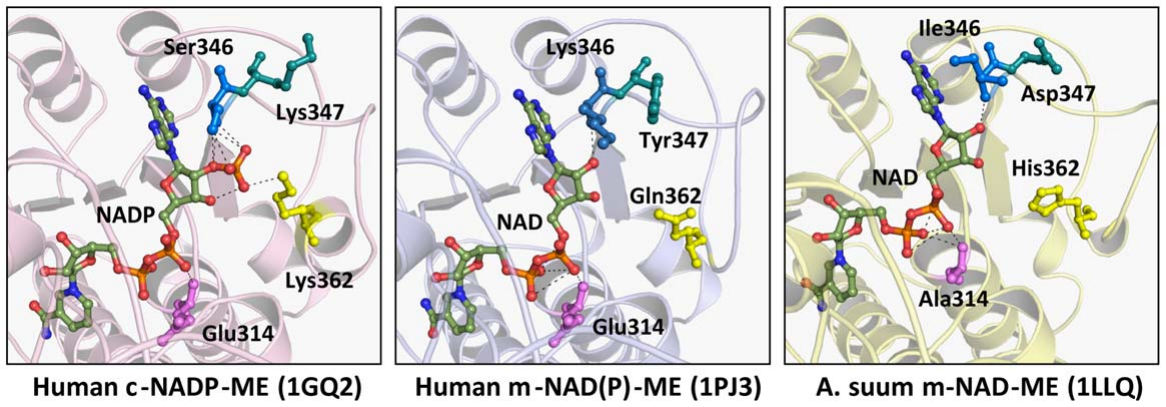
NAD^+^ or NADP^+^ cofactors in the nucleotide-binding pocket of the active center of malic enzyme. (**A**) The NADP-binding pocket of the pigeon c-NADP-ME (PDB code 1GQ2). (**B**) The NAD-binding pocket of the human m-NAD(P)-ME (PDB code 1PJ3). (**C**) The NAD-binding pocket of the *Ascaris suum* m-NAD-ME (PDB code 1LLQ). The gray dashed lines produced using PyMOL (DeLano Scientific LLC, San Carlos, CA) represent the polar contacts between the amino acid residues and NAD^+^ or NADP^+^.

Crystal structures of the nucleotide-binding sites of pigeon c-NADP-ME, human m-NAD(P)-ME and *Ascaris suum* m-NAD-ME are illustrated in Figure 3 and may be used as models for the human WT, S346K/K347Y/K362Q and E314A/S346I/K347D/K362H c-NADP-ME variants, respectively, to explain the molecular basis of the nucleotide-binding selectivity of malic enzyme.

At the nucleotide-binding site of pigeon c-NADP-ME, Lys362 and Ser346 interact directly with the 2′-phosphate of NADP^+^ (Figure 3A). Lys362 is ion-paired with the 2′-phosphate of NADP^+^ and is involved in the electrostatic network of Asp345 and Arg354; these interactions make the carboxylic side-chain of Asp345 deviate from the 2′-phosphate of NADP^+^, thereby reducing the repulsion between Asp345 and NADP^+^ and enhancing the affinity for NADP^+^ in the active site. Thus, the repulsive effect of Glu314 seems to be insignificant for NADP^+^ binding by this isoform because of its high affinity toward NADP^+^. Ser346 in c-NADP-ME is hydrogen-bonded to the 2′-phosphate of NADP^+^ and may assist in the binding of NADP^+^ (Figure 3A). Lys347 does not directly interact with NADP^+^. The positive charge of Lys347 may play a role in maintaining electrostatic balance in the nucleotide-binding site, thereby increasing the affinity for NADP^+^.

Lys362 in c-NADP-ME plays a major role in governing NADP^+^ specificity [30], [31], while Gln362 in human m-NAD(P)-ME mainly contributes to dual-cofactor specificity [14], [31] and Lys346 and Tyr347 are suggested to be collaborators that cooperatively confer cofactor selectivity. Therefore, the nucleotide-binding site of S346K/K347Y/K362Q c-NADP-ME may be similar to that of human m-NAD(P)-ME (Figure 3B); the reverse effect for this mutant enzyme on cofactor preference, switching from NADP^+^ to NAD^+^, was observed. However, the E314A/S346K/K347Y/K362Q c-NADP-ME showed greater favor for NAD^+^ than S346K/K347Y/K362Q c-NADP-ME. We have demonstrated that the E314A m-NAD(P)-ME has a smaller *K*_m,NAD_ value than WT m-NAD(P)-ME [18]. Considering the much smaller *K*_m_ of NADP^+^ for c-NADP-ME, the relatively higher *K*_m_ of NAD^+^ for m-NAD(P)-ME may be caused by the negative charge of Glu314.

The E314A/S346I/K347D/K362H c-NADP-ME, mimicking the *Ascaris suum* m-NAD-ME, was a NAD^+^-preferring and ATP-insensitive enzyme. The nucleotide-binding site of E314A/S346I/K347D/K362H c-NADP-ME may be similar to that of *Ascaris suum* m-NAD-ME (Figure 3C). Hydrophobic Ile346 and negatively-charged Asp347 have a significant repulsive effect on NADP^+^ and ATP. Previous work with *Ascaris suum* m-NAD-ME indicated that mutation of His362 to Lys did not cause a shift in cofactor specificity of the enzyme from NAD^+^ to NADP^+^ and that His362 in *Ascaris suum* m-NAD-ME is a second-layer residue in cofactor interaction [32]. According to our results here, we propose that replacement of Ile346 and Asp347 with Ser and Lys, respectively, in *Ascaris suum* m-NAD-ME may have an effect on changing the enzyme's cofactor preference to NADP^+^.

Considering these kinetic data collectively, we conclude that the quadruple mutants containing the E314A mutation display NAD^+^ specificity by significantly decreasing *K*_m,NAD_ and *K*_m,mal(NAD)_ and increasing *k*_cat,NAD_. These results indicate that in addition to residues 346, 347 and 362, Glu314 is also a determinant of nucleotide-binding affinity in malic enzyme.

## Materials and Methods

### Expression, purification, and characterization of human c-NADP-ME

The cDNA encoding c-NADP-ME was sub-cloned into the pET21b vector, which carries a C-terminal His6·Tag sequence. The *Escherichia coli* BL21(DE3) strain was transformed with the expression vector, which includes an inducible T7 promoter system. Enzyme overexpression was induced by 1.0 mM isopropyl β-D-1-thiogalactopyranoside (IPTG), and the overexpressed enzyme was purified using a HIS-Select™ Nickel Affinity Gel column (Sigma). The lysate-Ni-NTA mixture was washed with buffer (10 mM imidazole, 500 mM sodium chloride, 2 mM β-mercaptoethanol, and 30 mM Tris-HCl, pH 7.4) to remove unwanted proteins, and the c-NADP-ME was subsequently eluted with elution buffer (250 mM imidazole, 500 mM sodium chloride, 2 mM β-mercaptoethanol, and 30 mM Tris-HCl, pH 7.4).

The purified enzyme was buffer-exchanged and concentrated with an Amicon Ultra-15 centrifugal filter (Millipore Corp.) and preserved in 30 mM Tris-HCl (pH 7.4) with 2 mM β-mercaptoethanol. Enzyme purity was examined by SDS-PAGE, and protein concentration was determined by the Bradford method [33].

### Site-directed mutagenesis

Single (S346K, K347Y, and K362Q), double (S346K/K347Y, S346K/K362Q, and K347Y/K362Q), and triple (S346K/K347Y/K362Q) mutants were constructed by the QuikChange™ kit (Stratagene), using a plasmid containing the open reading frame encoding human c-NADP-ME as template for the mutagenesis. The PCR primers were as follows: 5′- CCAAGGAGCTGGA**GCG**GCTGCCCTAGGG-3′ for E314A; 5′-GATATGGCTGGTTGAT**AAA**AAAGGATTAATAGTTAAGGG-3′ for S346K; 5′-GGCTGGTTGATTCA**TAC**GGATTAATAGTTAAGGGACG-3′ for K347Y; 5′-GCTTCCTTAACACAAGAG**CAG**GAGAAGTTTGCCCATG-3′ for K362Q; 5′-GATATGGCTGGTTGATAAA**TAC**GGATTAATAGTTAAGGG-3′ for S346K/K347Y; 5′-GCTTCCTTAACACAAGAG**CAC**GAGAAGTTTGCCCATG-3′ for K362H; and 5′-GATATGGCTGGTTGAT**ATCGAC**GGATTAATAGTTAAGGGACG-3′ for S346I/K347D. The PCR reaction used *Pfu* DNA polymerase and was performed at 95°C for 30 sec, 55°C for 1 min, and 68°C for 2 min/kb of plasmid length for 16 cycles. The templates were digested with the *Dpn*I restriction enzyme and transformed into *E. coli* XL-1 cells. All mutation sites were confirmed by automated sequencing.

### Enzyme kinetic analysis

The malic enzyme reaction was assayed in a reaction buffer including saturating concentrations of L-malate, NAD^+^ or NADP^+^ and MgCl_2_ in 50 mM Tris-HCl (pH 7.4) in a total volume of 1 mL at 30°C. For *K*_m,mal_ determination, the concentrations of NAD(P)^+^ and MgCl_2_ were fixed at 1 mM and 10 mM, respectively, with various [malate]; for *K*_m,Mg_ determination, the concentrations of malate and NAD(P)^+^ were fixed at 15 mM and 1 mM, respectively, with various [Mg^2+^]; the malate and MgCl_2_ concentrations for *K*_m,NAD_ or *K*_m,NADP_ determination were fixed at 15 mM and 10 mM, respectively, with various [NAD^+^] or [NADP^+^]. The enzyme concentration used in these experiments was 5 µg/mL. Absorbance at 340 nm was continuously monitored in a UV/VIS Spectrophotometer Lambda 25 (Perkin Elmer, USA). An absorption coefficient of 6.22 mM^−1^ at 340 nm for NAD(P)H was used in the calculations. Apparent Michaelis constants for the substrate or the cofactor were determined by varying one substrate concentration near its *K*_m_ value while maintaining the other components constant at saturation levels. All calculations were conducted using the Sigma Plot 10.0 program (Jandel, San Rafael, CA). The *k*_cat_ value of c-NADP-ME was calculated by the following equation:
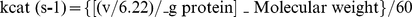
where *v* represents ΔA_340_/min, 6.22 is the millimolar absorption coefficient of NAD(P)H, 260,000 is the molecular weight of a tetramer of human c-NADP-ME and 60 is the number of seconds in one minute.

The ATP inhibition experiment was assayed with 50 mM Tris-HCl (pH 7.4), 40 mM malate (pH 7.4), 10 mM MgCl_2_, and 1.0 mM NAD^+^ or NADP^+^ (pH 7.4) at a series of ATP concentrations, ranging from 0 to 3 mM. The *K*_i_ value of all enzymes were assayed with the reaction buffer consisting of 50 mM Tris-HCl (pH 7.4), 20 mM malate (pH 7.4), and 10 mM MgCl_2_ at a series of ATP concentrations around its *K*_i_ value and at a series of NAD^+^ or NADP^+^ (pH 7.4) concentrations around its *K*_m_ value. The following equation was globally fitted to the total data set, which describes a competitive inhibition pattern:
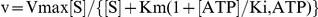
where *v* is the observed initial velocity, *V*_max_ is the maximum rate of the reaction, *K*_m_ is the Michaelis constant for the substrate, and *K*_i,ATP_ is the inhibition constant for ATP.
